# Heparin-binding protein in lower airway samples as a biomarker for pneumonia

**DOI:** 10.1186/s12931-021-01764-2

**Published:** 2021-06-08

**Authors:** Magnus Paulsson, Louise Thelaus, Kristian Riesbeck, Ingemar Qvarfordt, Margaretha E. Smith, Anders Lindén, Adam Linder

**Affiliations:** 1 Department of Infectious Diseases, Skåne University Hospital, Lund, Sweden; 2 Division of Infection Medicine, Department of Clinical Sciences, Faculty of Medicine, Lund University, BMC B14, SE221 85 Lund, Sweden; 3 Clinical Microbiology, Department of Translational Medicine, Faculty of Medicine, Lund University, Malmö, Sweden; 4 Department of Internal Medicine and Clinical Nutrition, Institute of Medicine, Sahlgrenska Academy, University of Gothenburg, Gothenburg, Sweden; 5 Unit for Lung and Airway Research, Institute of Environmental Medicine, Karolinska Institutet, Stockholm, Sweden; 6 Karolinska Severe COPD Center, Department of Respiratory Medicine and Allergy, Karolinska University Hospital, Stockholm, Sweden

**Keywords:** Pneumonia, Critical Care, Neutrophil Biology, Assisted Ventilation, Biomarkers, Bronchoalveolar lavage

## Abstract

**Objectives:**

Ventilator-associated pneumonia (VAP) is difficult to diagnose using clinical criteria and no biomarkers have yet been proved to be sufficiently accurate. The use of the neutrophil-derived Heparin-binding protein (HBP) as a biomarker for pneumonia was investigated in this exploratory case–control study in two intensive care units at a tertiary referral hospital.

**Methods:**

Patients with clinical signs of pneumonia were recruited and bronchoalveolar lavage fluid (BALF) or bronchial wash (BW) samples were collected. Mechanically ventilated and lung healthy subjects were recruited as controls. HBP was measured with enzyme-linked immunosorbent assay.

**Results:**

BALF was collected from 14 patients with pneumonia and 14 healthy controls. Median HBP in BALF pneumonia samples was 14,690 ng/ml and controls 16.2 ng/ml (*p* < 0.0001). BW was collected from 10 pneumonia patients and 10 mechanically ventilated controls. Median HBP in BW pneumonia was 9002 ng/ml and controls 7.6 ng/ml (*p* < 0.0001).

**Conclusions:**

These data indicate that HBP concentrations is significantly higher in lower airway samples from patients with pneumonia than control subjects and is a potentially useful biomarker for diagnosis of VAP.

## Introduction

Ventilator-associated pneumonia (VAP), hospital-acquired pneumonia (HAP) and community-acquired pneumonia are conditions diagnosed based on clinical criteria and cultures from lower airway samples (LAS) [1]. The addition of biomarkers in plasma or bronchoalveolar lavage fluid (BALF) have not yet been proved to add substantial clinical value and poor biomarkers increase the risk of incorrect diagnosis, leading to unnecessary antibiotic treatment or increased time to correct diagnosis. The ATS/IDSA do not recommend biomarker-guided HAP/VAP diagnosis as the sensitivity and specificity in published reports failed to exceed 90% [1]. Still, semiquantitative cultures on respiratory samples constitute gold-standard but these cultures are time consuming and can be biased by previous antibiotic treatment or presence of unculturable pathogens. Since VAP significantly increases mortality, a biomarker that accurately identifies VAP would be highly valuable [2].

Heparin-binding protein (HBP) is present in azurophilic granules and secretory vesicles of neutrophils and is released by activated neutrophils. Its known properties include antimicrobial effects, monocyte and macrophage activation, and particularly induction of vascular leakage [3]. Several studies have successfully evaluated plasma HBP as a biomarker for prognosticating organ dysfunction in sepsis and septic shock and there is evidence that HBP in BALF from patients with lung allografts can detect pulmonary infection with a cut-off value of 150 ng/mL [4]. In addition, the severity of bronchiectasis as well as exacerbations of cystic fibrosis correlate with sputum HBP [5, 6]. In this exploratory study, we evaluated the biomarker potential of HBP in LAS from patients with pneumonia.

## Materials and methods

Patients displaying clinical symptoms of pneumonia (temperature > 38 °C or < 36 °C, purulent tracheal aspirate or decreased oxygen saturation) and radiological signs (new infiltrate on Chest X-ray) were recruited at the Departments of Infectious Diseases or Anesthesiology and Intensive Care at Skåne University Hospital (Malmö, Sweden) from 2015 to 2017. BALF (3 × 50 ml sterile phosphate-buffered saline, PBS) was collected from the first 14 recruited patients (“Pneumonia 2016”), while BW (2 × 10 ml PBS) was collected from the following 10 patients (“Pneumonia 2017”). In both patient groups, the most affected lung segment was identified based on appearance at the time of bronchoscopy and chosen for sampling, as described [7]. Mechanically ventilated and endotracheally intubated control subjects for BW (*n* = 10, “BW control”) were recruited to avoid the potentially confounding influence of mechanical ventilation on HBP concentrations. These control subjects were orthopedic patients without pulmonary disorders being planned for back surgery. To establish appropriate control samples for BALF, we utilized samples from unexposed healthy volunteers (*n* = 14), who were recruited to the Section of Respiratory Medicine, Sahlgrenska University Hospital (Gothenburg, Sweden) for a previously published study on the local effects of endotoxin exposure [8]. Baseline data of all included study subjects are summarized in Table 1.

**Table 1:** Baseline characteristics of all included subjects

Variable	Study group			
	BALF “Pneumonia 2016”	BALF control	BW “Pneumonia 2017”	BW control
Number of subjects	14	14	10	10
Males	8 (57.1)	7 (50.0)	8 (80.0)	4 (40.0)
Age (years)	74 (60.8–82.0)	23.5 (22–24)	66 (58.5–68.8)	55 (39–59)
Current smoker	0 (0.0)	0 (0.0)	2 (20.0)	0 (0.0)
COPD	4 (28.6)	0 (0.0)	3 (30.0)	0 (0.0)
Other pulmonary diseases	1 (7.1)	0 (0.0)	0 (0.0)	0 (0.0)
Diabetes mellitus	4 (28.6)	0 (0.0)	1 (10.0)	1 (10.0)
Cardiovascular disease	5 (35.7)	0 (0.0)	3 (30.0)	2 (20.0)
Non-pulmonary malignancy	5 (35.7)	0 (0.0)	2 (20.0)	0 (0.0)
Radiographic lung infiltrate	11 (78.6)	NA	6 (60.0)	0 (0.0)
Purulent sputum	8 (57.1)	0 (0.0)	3 (30.0)	0 (0.0)
Temp > 38 °C within the last 24 h	12 (85.7)	0 (0.0)	6 (60.0)	0 (0.0)
Days with ventilator	5 (4.0–6.0)	NA	2.5 (1.8–6.5)	0.5 (0–1.0)
Arterial oxygen saturation (%)	93.5 (92.0–94.0)	98 (98.0–99.0)	95.5 (93.3–98.0)	97 (96–98)
Plasma CRP (mg/l)	69.5 (23.5–142.8)	NA	89.5 (53.3–188.0)	3.1 (1.3–10.9)
Blood leukocytes (10^9 cells/l)	10.6 (9.0–15.0)	6.4 (5.4–7.8)	10.3 (7.4–16.7)	6.9 (5.7–8.7)
Blood neutrophils (10^9 cells/l)	9.1 (8.7–13.2)	3.5 (2.7–4.0)	5.7 (5.6–9.9)	3.4 (3.3–5.8)
Gram-positive PPM	5 (35.7)	NA	3 (30.0)	0 (0.0)
Gram-negative PPM	7 (50.0)	NA	6 (60.0)	0 (0.0)
Viral PMM	1 (7.1)	NA	0 (0.0)	0 (0.0)
Fungal PMM	1 (7.1)	NA	0 (0.0)	0 (0.0)
Antibiotic treatment	14 (100.0)	0 (0.0)	10 (100.0)	6 (60.0)
Systemic steroid treatment (= > 10 mg prednisolon)	3 (21.4)	0 (0.0)	2 (20.0)	0 (0.0)
Inhalation steroid treatment	3 (21.4)	0 (0.0)	6 (60.0)	0 (0.0)
Other immunosuppression	1 (7.1)	0 (0.0)	1 (10.0)	0 (0.0)

Data are presented as median (interquartile range) or *n* (%), unless otherwise stated. *BW* bronchial wash, *BALF* bronchoalveolar lavage fluid, *COPD* chronic obstructive pulmonary disease, *CRP* C-reactive protein, *PPM* potentially pathogenic microorganism;

We quantified HBP in BALF or bronchial wash (BW) from patients with pneumonia (*n* = 24) and from control subjects (*n* = 24) using a commercial ELISA kit (Axis-Shield Diagnostics, Dundee, United Kingdom) in accordance to the manufacturer’s instructions.

Statistical analysis was made using Prism software (Graphpad v8.4.3, San Diego, CA). Two-tailed *p*-values were calculated using Mann–Whitney’s test. A receiver-operating characteristic (ROC) curve was calculated with 95% confidence interval (CI).

## Results

**Fig. 1:**
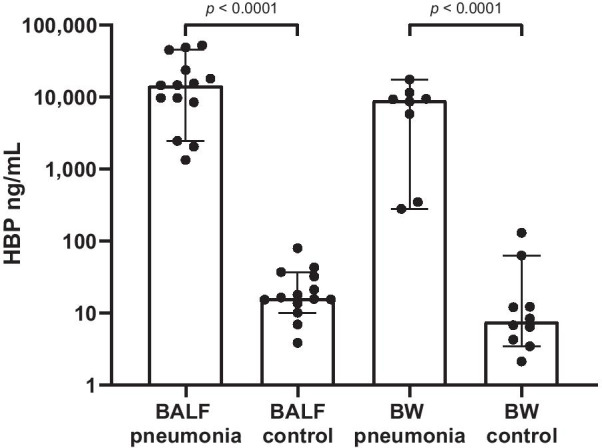
Concentrations of Heparin-binding protein (HBP) were measured in bronchoalveolar lavage fluid (BALF) and bronchial wash (BW) samples from patients with pneumonia and from healthy control subjects. The median HBP in BALF “Pneumonia 2016” samples was 14,690 ng/ml and BALF control 16.2 ng/ml (*p* < 0.0001). The median HBP in BW “Pneumonia 2017” samples was 9,002 ng/ml and BW control median was 7.6 ng/ml (*p* < 0.0001). Bar graph show median values and 95% confidence intervals. Each dot represents one study subject. Statistical evaluations were made with Mann–Whitney test. *P*-values are indicated on the graph

The concentration of HBP was significantly increased in samples from patients with pneumonia compared those from control subjects, whether collected as BALF or as BW (Fig. 1). Two “Pneumonia 2017” subjects were excluded from further analysis because of negative cultures, all other samples contained bacterial pathogens. All control subjects had HBP concentrations below the previously proposed cut-off of 150 ng/ml and all pneumonia patients displayed concentrations above 150 ng/ml. The two excluded subjects both had HBP values below 150 ng/ml. We observed no statistically significant difference in HBP concentrations between BALF and BW samples. Given this, a ROC curve was calculated using pooled samples from both pneumonia patients and control subjects. Best diagnostic accuracy was achieved using a cut-off of 206 ng/ml, that yielded a sensitivity to detect pneumonia of 100% (95% CI = 85.1 – 100%) and a specificity of 100% (95% CI = 86.2 – 100%).

## Discussion

The usefulness of HBP as biomarker for pneumonia depends on its accuracy in differentiating pneumonia from other diagnoses, ease of sample collection and time from sampling to results. Importantly, HBP can be analyzed using a point-of-care device in less than 30 min, a fact that enables HBP in LAS to influence the decision to start antibiotic therapy. The recent VAPrapid2 trial investigated if IL-1β and IL-8 in BALF were useful in an antibiotic stewardship design [9]. However, antibiotic prescription remained unchanged, which was partly attributed to reluctance for collecting BALF in critically ill patients. In view of this, we included a BW cohort and found no significant difference in HBP concentrations between BALF and BW. Although not as accessible as blood samples, BW samples are specific for the conditions in the lungs and the smaller lavage volumes of BW are less likely to cause adverse effects than BAL and may be more tolerable for the clinician. In addition, the BW control group was mechanically ventilated and better matched to the pneumonia patients in terms of age. Yet, the HBP values in the BW control group were similar to those in the BALF control group and indicated no increase in HBP related to mechanical ventilation.

We did not normalize HBP concentrations to urea or return volume, because normalization may confound the results and omitting normalization is in line with current recommendations and imitates the clinical setting [10]. Instead, our sampling protocol was standardized with BALF collected using 3 × 50 ml lavage fluid and BW collected with 2 × 10 ml. Yet, we obtained very clear-cut results. The latter and the fact that we explored a limited study sample, supports the idea that HBP possesses substantial potential as a robust biomarker for clinical use. Nevertheless, a larger study sample would allow independent ROC analysis of each material, so larger and prospective cohort studies in critically ill patients are warranted in the near future to verify the diagnostic accuracy and the optimal positive test cut-off.

## Conclusions

In conclusion, this exploratory study forward evidence that the median HBP concentration in LAS is enhanced around a 1000-fold in patients with pneumonia. This indicates that HBP in LAS is a potential biomarker that may be added to current diagnostic tools for VAP.

## Data Availability

The datasets used and/or analysed during the current study are available from the corresponding author on reasonable request.

## Abbreviations

BALF: Bronchoalveolar lavage fluid
BW: Bronchial wash
COPD: Chronic obstructive pulmonary disease
CRP: C-reactive protein
HAP: Hospital-acquired pneumonia
HBP: Heparin-binding protein
LAS: Lower airway samples
PBS: Phosphate-buffered saline
PPM: Potentially pathogenic microorganism
ROC: Receiver-operating characteristic
VAP: Ventilator-associated pneumonia
